# *KRAS* status is related to histological phenotype in gastric cancer: results from a large multicentre study

**DOI:** 10.1007/s10120-019-00972-6

**Published:** 2019-05-20

**Authors:** Lindsay C. Hewitt, Yuichi Saito, Tan Wang, Yoko Matsuda, Jan Oosting, Arnaldo N. S. Silva, Hayley L. Slaney, Veerle Melotte, Gordon Hutchins, Patrick Tan, Takaki Yoshikawa, Tomio Arai, Heike I. Grabsch

**Affiliations:** 1 Department of Pathology, GROW School for Oncology and Developmental Biology, Maastricht University Medical Center+, P. Debyelaan 25, 6229 HX Maastricht, The Netherlands; 2 Division of Pathology and Data Analytics, Leeds Institute of Medical Research at St. James’s, University of Leeds, Leeds, UK; 3 Department of Pathology, Tokyo Metropolitan Geriatric Hospital and Institute of Gerontology, Tokyo, Japan; 4 Department of Comprehensive Pathology, Tokyo Medical and Dental University, Tokyo, Japan; 5 Department of Pathology, Leiden University Medical Center, Leiden, The Netherlands; 6 Department of Clinical Genetics, Erasmus University Medical Center, University of Rotterdam, Rotterdam, The Netherlands; 7 Duke-NUS Medical School, Singapore, Singapore; 8 Department of Gastric Surgery, National Cancer Center Hospital, Tokyo, Japan; 9 Department of Gastrointestinal Surgery, Kanagawa Cancer Center Hospital, Yokohama, Japan

**Keywords:** Gastric cancer, *KRAS*, Mutation, Amplification, Histological phenotype

## Abstract

**Background:**

Gastric cancer (GC) is histologically a very heterogeneous disease, and the temporal development of different histological phenotypes remains unclear. Recent studies in lung and ovarian cancer suggest that *KRAS* activation (*KRAS*act) can influence histological phenotype. *KRAS*act likely results from *KRAS* mutation (*KRAS*mut) or *KRAS* amplification (*KRAS*amp). The aim of the study was to investigate whether *KRAS*mut and/or *KRAS*amp are related to the histological phenotype in GC.

**Methods:**

Digitized haematoxylin/eosin-stained slides from 1282 GC resection specimens were classified according to Japanese Gastric Cancer Association (JGCA) and the Lauren classification by at least two observers. The relationship between *KRAS* status, predominant histological phenotype and clinicopathological variables was assessed.

**Results:**

*KRAS*mut and *KRAS*amp were found in 68 (5%) and 47 (7%) GCs, respectively. Within the *KRAS*mut and *KRAS*amp cases, the most frequent GC histological phenotype was moderately differentiated tubular 2 (tub2) type (*KRAS*mut: *n* = 27, 40%; *KRAS*amp: *n* = 21, 46%) or intestinal type (*KRAS*mut: *n* = 41, 61%; *KRAS*amp: *n* = 23, 50%). Comparing individual histological subtypes, mucinous carcinoma displayed the highest frequency of *KRAS*mut (JGCA: *n* = 6, 12%, *p* = 0.012; Lauren: *n* = 6, 12%, *p* = 0.013), and *KRAS*amp was more frequently found in poorly differentiated solid type (*n* = 12, 10%, *p* = 0.267) or indeterminate type (*n* = 12, 10%, *p* = 0.480) GC. 724 GCs (57%) had intratumour morphological heterogeneity.

**Conclusions:**

This is the largest GC study investigating *KRAS* status and histological phenotype. We identified a relationship between *KRAS*mut and mucinous phenotype. The high level of intratumour morphological heterogeneity could reflect *KRAS*mut heterogeneity, which may explain the failure of anti-EGFR therapy in GC.

**Electronic supplementary material:**

The online version of this article (10.1007/s10120-019-00972-6) contains supplementary material, which is available to authorized users.

## Introduction

Gastric cancer (GC) is histologically a very heterogeneous disease, and this is reflected in the numerous proposed histological classification schemes [1]. The temporal development of different histological phenotypes in GC remains unclear. Recent studies suggest that Kirsten Rat Sarcoma Viral Oncogene Homolog *(KRAS)* activation and downstream signalling can impact on the properties and functions of the tumour microenvironment [2], and thus may influence histological phenotype. Likely mechanisms of *KRAS* activation include *KRAS* mutation (*KRAS*mut) and *KRAS* amplification (*KRAS*amp) [3].

Mutations in *KRAS* have been identified in many human cancers and result in the constitutive activation of *KRAS* and the receptor tyrosine kinase (RTK) pathway [4]. The frequency of *KRAS*mut is variable across different cancer types, with the highest frequency in pancreatic cancer (90%) followed by colon (34.6%), lung (16.5%) and ovarian (11%) cancer and the lowest frequencies in cervical (6.6%), prostate (5%) and oesophageal cancer (2%) [5]. In a review of the literature we identified, on average, only 6.5% of GC have a *KRAS*mut [6]. In colorectal cancer, routine testing for *KRAS*mut is now implemented as a predictor of response to anti-epidermal growth factor receptor (EGFR) therapy [7].

Several studies have demonstrated a relationship between *KRAS*mut status and histological phenotype in lung and ovarian cancer. In the subgroup of invasive mucinous adenocarcinoma of the lung, *KRAS* is mutated in up to 86% of cases [8]. In ovarian cancer, *KRAS*mut has been identified in almost all cases with a mucinous histological phenotype [9]. The relationship between *KRAS*mut status and histological phenotype in GC remains to be clarified [6].

The reported frequency of *KRAS*amp is 1–9% in GC [10–16]. There are no reports of a relationship between *KRAS* DNA copy number and histological phenotype in other cancer types and in GC it has not been investigated in a large study. There is increasing recognition of the clinical importance of *KRAS*amp in GC. *KRAS*amp is also associated with a worse survival [3, 10, 12], whereas *KRAS*mut do not appear to influence survival of GC patients [17].

Recently, image analysis on lung cancer haematoxylin and eosin (H&E) stained sections using deep learning was predictive of mutation status [18], thus suggesting that morphological phenotype is reflective of molecular phenotype. Investigating the relationship between *KRAS* activation by *KRAS*mut and/or *KRAS*amp and histological phenotype may provide some insight into gastric adenoma–carcinoma sequence progression and the origin of histological heterogeneity. Based on the studies in lung and ovarian cancer, we hypothesise that *KRAS* activation influences histological phenotype and is associated with a mucinous phenotype in GC. This would suggest that *KRAS* activation is an early event in GC, occurring before the phenotype is determined.

The aim of this multicentre GC study was to investigate the relationship of *KRAS* activation status (*KRAS*mut and/or *KRAS*amp) with the histological phenotype in a large series of GCs from UK, Japan and The Cancer Genome Atlas (TCGA). In addition, the relationship between *KRAS* status, clinicopathological variables, survival and microsatellite instability status was assessed.

## Material and methods

### Patients

#### Kanagawa Cancer Center Hospital (KCCH), Yokohama, Japan

This cohort included 250 patients with TNM stage II/III GC who underwent potentially curative surgery at Kanagawa Cancer Center Hospital (Yokohama, Japan) between 2001 and 2010. One hundred and six (43%) patients were treated with surgery alone, 108 (43%), 22 (9%), 14 (6%) patients received S-1, tegafur–uracil or S-1 combined with other cytotoxic drug therapy, respectively. Demographical, clinical and pathological data were retrieved from hospital records. The study was approved by the Local Research Ethics Committee.

#### Leeds Teaching Hospitals NHS Trust (LTHT), Leeds, UK

This cohort included 277 patients with GC who underwent potentially curative surgery at the Department of Surgery, Leeds General Infirmary (Leeds, UK), between 1970 and 2004. Seven (3%) patients were treated by chemotherapy followed by surgery and the remaining 270 (98%) by surgery alone. Clinical and pathological data were retrieved from histopathology reports, electronic patient hospital records and the Northern and Yorkshire Cancer Registry. The study was approved by the Local Research Ethics Committee (LREC no. CA01/122).

#### The Cancer Genome Atlas

The TCGA stomach adenocarcinoma (STAD) clinicopathological and molecular dataset of 295 patients was obtained from the publically available TCGA database portal [19].

#### Tokyo Metropolitan Geriatric Hospital and Institute of Gerontology (TMGH), Tokyo, Japan

This cohort included 420 patients with 460 GC who were treated by surgery in the Tokyo Metropolitan Geriatric Hospital between 2000 and 2008. Three hundred and eighty patients had single carcinoma, and 36 had 2 or more carcinomas. Patients with Lynch syndrome were excluded from the current study. None of the patients underwent neoadjuvant chemotherapy. Histopathological examination and medical research were performed with informed written consent by the patients, and this work was approved by the ethics committee of the Tokyo Metropolitan Geriatric Hospital (#230,225, R16-23).

#### Histopathological classification

pT and pN stages were reported according to 7th edition of the UICC TNM classification for GC [20].

In all cohorts, H&E stained formalin fixed paraffin embedded (FFPE) tissue sections from the resection specimens were reviewed. In the KCCH and LTHT cohorts, H&E stained slides were scanned at 40 × magnification using an Aperio AT2 scanner for review. In the TCGA cohort, H&E stained slides were viewed online using the cancer digital slide archive (https://cancer.digitalslidearchive.net/). In the TMGH cohort, classification was performed using the glass slides.

Histological classification according to JGCA scheme was performed [21]. Mucinous carcinoma were defined as tumour cells located in mucinous pools comprising an area greater than 50% of the total tumour. GC were classified as signet-ring cell carcinoma when signet-ring cells were present in more than 50% of the tumour volume. In cases where more than one histological phenotype was identified, the most predominant phenotype was recorded, and these GCs were categorised as heterogeneous. JGCA classification was converted to Lauren classification [22] according to Table 1. As there is no Lauren classification for mucinous GC, we retained mucinous carcinomas as a separate category to distinguish them from other histological types.

**Table 1:** Japanese Gastric Cancer Association histological classification of common types of gastric cancers in relation to Lauren classification

Histological classification	
Lauren	Japanese Gastric Cancer Association (JGCA)
Intestinal	Differentiated: Papillary adenocarcinoma (pap) Tubular adenocarcinoma (tub) Well-differentiated (tub1) Moderately differentiated (tub2)
Diffuse	Undifferentiated: Poorly differentiated adenocarcinoma (por) Non-solid type (por2) Signet-ring carcinoma (sig)
Mucinous	Differentiated/undifferentiated: Mucinous adenocarcinoma (muc)
Indeterminate	Undifferentiated: Poorly differentiated adenocarcinoma (por) Solid type (por1)

Table created after personal communication with H. Grabsch, March 12, 2019

#### DNA extraction

The area with the highest tumour cell density was identified on H&E stained sections and the whole tumour area, irrespective of subregions with different histological phenotypes was microdissected after staining with Shandon instant haematoxylin (Thermo Scientific, Cheshire, UK) using a sterile surgical blade. Tumour DNA from FFPE material was extracted from KCCH and LTHT GCs using the QIAmp DNA Micro Kit (Qiagen, Hilden, Germany) as previously described [23]. DNA concentration was measured by ND-100 Spectrophotometer (Labtech International) and samples were diluted using Tris-EDTA buffer. In the TMGH cohort, DNA was extracted using a phenol–chloroform procedure as described previously [24].

#### *KRAS* gene copy number and data analyses

*KRAS* copy number status was investigated in KCCH, LTHT and TCGA cohorts.

In the KCCH and LTHT cohort, *KRAS* gene copy number was determined by multiplex ligation-dependent probe amplification (MLPA) using the Salsa-FAM-labeled MLPA reagent kit and probemix P458-A1 or the updated version -B1 (MRC Holland, Amsterdam, The Netherlands) as previously described [25]. For further details on the *KRAS* probes included in this probemix see Supplementary Table 1. Fragment analysis of the MLPA reaction product was performed using capillary electrophoresis ABI-3130 XL (Applied Biosystems, California, USA) as previously described [25]. Failed experiments were repeated at least twice before a case was finally excluded from the analyses.

*KRAS* DNA copy number data from 237 KCCH GC has been previously published [25], but was re-analysed using a different methodology in the current study. The output files (FSA files) from the sequencer were initially imported into Coffalyser.net for fragment analysis and results were exported as csv files. Subsequent analyses were performed using the MLPAInter method, as previously described [26], implemented in R. Samples were normalised per batch using reference samples processed in each batch. Quality control was performed to exclude samples with low overall intensity, with a large difference in intensity between short probes and long probes, with low intensity of denaturation controls, or high within gene variation, defined as the average of the standard deviation of log-transformed values. Final values were calculated by averaging the peak height of each probe and then averaging the results of replicates. Copy number thresholds were set based on previously published studies [25, 27, 28], with a DNA copy number > 1.31 categorised as amplification. This analysis was performed separately for KCCH and LTHT cohorts.

In TCGA, *KRAS*amp were determined by array-based somatic copy number analysis [29]. Level 3 copy number segmentation data was downloaded from the TCGA data portal [19] and used to estimate copy number for *KRAS*. Based on previous studies, a LogRatio > 0.4 was categorised as amplification [30].

#### *KRAS* mutation status

*KRAS*mut data from a previous study were available for 230 KCCH and 275 LTHT GC patients [17]. *KRAS*mut testing was performed on an additional 12 KCCH GCs as previously described [17]. In TCGA, *KRAS*mut status was determined by whole-exome sequencing [29] and results were downloaded from the TCGA database portal [19] for 289 patients. In the TMGH cohort, *KRAS* (codon 12 and 13) was examined by polymerase chain reaction-restriction fragment length polymorphism (PCR-RFLP), using primers and methods previously described [31, 32].

#### Microsatellite instability (MSI) status

Immunohistochemistry of DNA mismatch repair proteins were used as a surrogate marker of MSI status. Results for MLH1, MSH2, MSH6 and PMS2 were available from 230 KCCH GCs, and MLH1 and MSH2 from 253 LTHT GCs from a previous study [17]. MLH1, MSH2, MSH6 and PMS2 immunohistochemistry was performed on additional 13 GCs from the KCCH cohort for this study, as previously described [17].

In TCGA, MSI was determined by a DNA based MSI-Mono-Derived-Dinucleotide Assay using four mononucleotide repeat loci and three dinucleotide repeat loci using a multiplex fluorescent-labeled PCR and capillary electrophoresis [29]. Results were obtained from the TCGA database portal [19] for 295 GC patients. MSI-low GCs were grouped with microsatellite stable (MSS) GCs for further analyses following current guidelines [33].

In the TMGH cohort, mononucleotide repeats *BAT25* and *BAT26* were investigated, as previously described [34–36] and GC were classified as MSS or MSI.

### Statistical analyses

All statistical analyses were performed using SPSS software version 23 (SPSS Inc., Chicago, III). The relationship between *KRAS*mut or *KRAS*amp and clinicopathological variables (age, gender, depth of invasion (pT), lymph node status (pN), TNM stage, Lauren classification [22], JGCA classification [21], MSI status and morphological heterogeneity status) was assessed using Chi-squared or Fisher’s exact test. The relationship between *KRAS*mut and survival in LTHT and KCCH cohorts has been published previously [17]. Combining all cohorts, the relationship between *KRAS*mut or *KRAS*amp and 5-year overall survival was analysed using the Kaplan–Meier method and differences were assessed using the log rank test. A *p* value of < 0.05 was considered significant.

## Results

### Patient characteristics

The median (range) age of GC patients was as follows; KCCH: 65 years (35–85 years), LTHT: 72 years (14–96 years), TCGA: 68 years (35–90 years), TMGH: 78 years (51–96 years). For a summary of other patient clinicopathological variables in each cohort see Table 2.

**Table 2:** Comparison of clinicopathological variables in each gastric cancer cohort

	Total (*n*) 1282	Total (%)	KCCH (*n*) 250	KCCH (%) 20	LTHT (*n*) 277	LTHT (%) 22	TCGA (*n*) 295	TCGA (%) 23	TMGH (*n*) 460	TMGH (%) 36
Age (years)										
< 65	343	27	122	49	78	28	123	42	20	4
≥ 65	936	73	128	51	199	72	169	58	440	96
Gender										
Male	769	60	175	70	164	59	182	62	248	54
Female	513	40	75	30	113	41	113	38	212	46
T stage										
pT1	272	21	6	2	20	7	11	4	235	51
pT2	138	11	43	17	24	9	44	15	27	6
pT3	350	28	34	14	79	29	155	54	82	18
pT4	512	40	167	67	154	56	75	26	116	25
N stage										
pN0	489	39	42	17	87	31	97	34	263	57
pN1	247	19	58	23	52	19	64	23	73	16
pN2	229	18	67	27	54	20	58	20	50	11
pN3	306	24	83	33	84	30	65	23	74	16
TNM stage										
I	307	24	0		34	12	32	12	241	53
II	384	30	97	39	81	29	116	42	90	20
III	507	40	153	61	151	55	111	40	92	20
IV	67	5	0		11	4	20	7	36	8
Lauren classification										
Diffuse	293	23	83	34	60	22	73	25	77	17
Intestinal	698	55	103	42	145	54	156	53	294	64
Mucinous	51	4	10	4	10	4	20	7	11	2
Indeterminate	229	18	51	21	56	21	44	15	78	17
JGCA classification										
Pap	71	6	5	2	9	3	17	6	40	9
Tub1	219	17	18	7	55	20	23	8	123	27
Tub2	408	32	80	32	81	30	116	40	131	29
Por1	229	18	51	21	56	21	44	15	78	17
Por2	227	18	63	26	52	19	71	24	41	9
Sig	66	5	20	8	8	3	2	1	36	8
Muc	51	4	10	4	10	4	20	7	11	2
Morphological heterogeneity										
Homogenous	542	43	102	42	82	30	185	63	173	38
Heterogeneous	724	57	140	58	189	70	108	37	287	62
*KRAS* mutation status										
Mutant	68	5	10	4	16	6	28	10	14	3
Wild type	1198	95	232	96	259	94	261	90	446	97
*KRAS* gene copy number										
Amplified	47	7	12	6	17	8	18	8	-	-
Other	602	93	196	94	199	92	207	92	-	-
Microsatellite instability status										
MSI	199	16	23	9	31	12	64	22	81	18
MSS	1057	84	223	91	224	88	231	78	379	82

Some variables do not add up to 1282 due to missing data

*JGCA* Japanese Gastric Cancer Association, *Pap* papillary adenocarcinoma, *Tub1* well-differentiated tubular adenocarcinoma, *Tub2* moderately differentiated tubular adenocarcinoma, *Por1* poorly differentiated adenocarcinoma solid type, *Por2* poorly differentiated adenocarcinoma non-solid type, *Sig* signet-ring cell carcinoma, *Muc* mucinous adenocarcinoma, *MSI* microsatellite instable, *MSS* microsatellite stable, *KCCH* Kanagawa Cancer Center Hospital, *LTHT* Leeds Teaching Hospital Trust, *TCGA* The Cancer Genome Atlas, *TMGH *Tokyo Metropolitan Geriatric Hospital and Institute of Gerontology

### Histological classification of gastric cancer

Histological classification was available for 1271 GCs. Using the JGCA classification, the most predominant phenotype was moderately differentiated tubular  [tub2] (*n* = 408, 32%), followed by poorly differentiated solid type [por1] (*n* = 229, 18%), poorly differentiated non-solid type [por2] (*n* = 227, 18%), well-differentiated tubular [tub1] (*n* = 219, 17%), papillary [pap] (*n* = 71, 6%), signet-ring cell [sig] (*n* = 66, 5%) and mucinous [muc] (*n* = 51, 4%). According to Lauren classification, 293 (23%) GCs were classified as diffuse type, 698 (55%) as intestinal type, 51 (4%) as mucinous and 229 (18%) as indeterminate. Seven hundred and twenty-four GCs (57%) had intratumour morphological heterogeneity (see Table 2).

### *KRAS* mutation status and relationship with clinicopathological variables

**Fig. 1:**
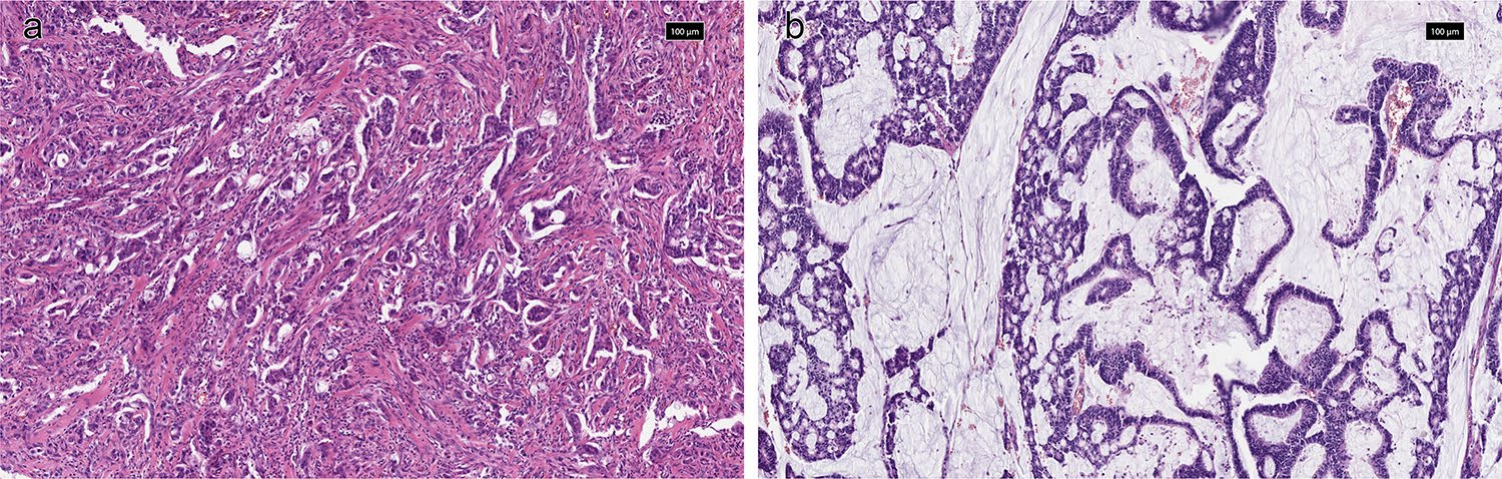
Example of *KRAS* mutated GC with **a** moderately differentiated tubular (tub2) phenotype and **b** mucinous (muc) phenotype

**Fig. 2:**
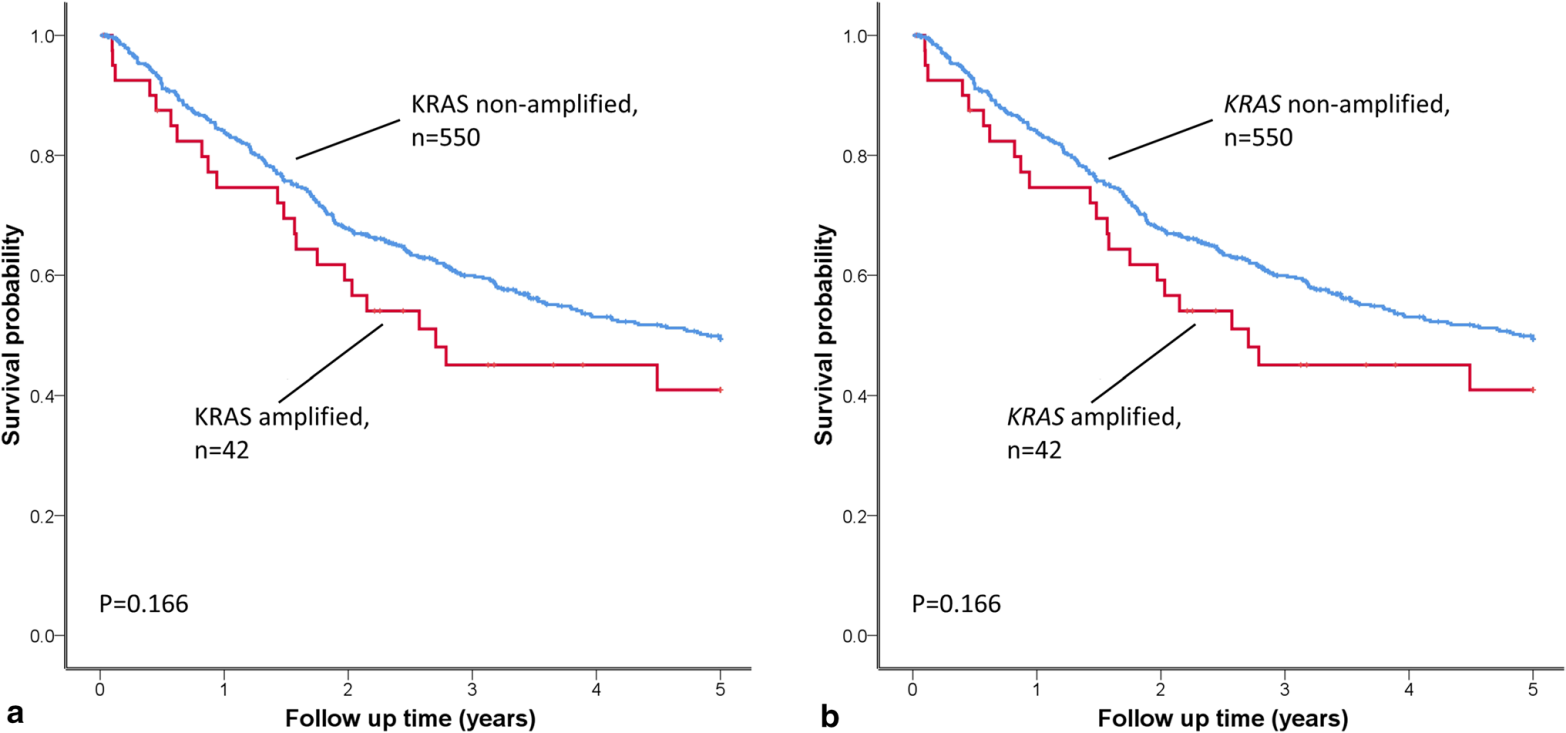
Kaplan–Meier plots showing probability of overall survival in GC patients stratified by *KRAS* gene activation status. **a** Kaplan–Meier survival analysis showed no difference in survival when patients were stratified by *KRAS* mutation status. **b** Kaplan–Meier survival analysis showed no difference in survival when patients were stratified by *KRAS* amplification status

*KRAS*mut status was available from 1266 GCs (KCCH *n* = 242; LTHT *n* = 275; TCGA *n* = 289, TMGH *n* = 460). In total, 68 (5%) GCs were *KRAS* mutant, with the highest frequency of *KRAS*mut in the TCGA cohort (10%) and lowest frequency in the TMGH cohort (3%), see Table 2. Within the *KRAS*mut GC, the most frequent histological phenotype was intestinal type (*n* = 41, 61%) or tub2 (*n* = 27, 40%) by Lauren and JGCA classification, respectively (see Fig. 1a). Comparing individual histological subtypes, mucinous phenotype displayed the highest frequency of *KRAS*mut by Lauren (*p* = 0.013) and JGCA (*p* = 0.012) classification, respectively (see Fig. 1b). *KRAS*mut was more frequent in MSI GC (*p* < 0.001). For the comparison of *KRAS*mut status and other clinicopathological variables, see Table 3. The 5-year overall survival rate in patients with *KRAS*mut or *KRAS* wild type GC was 63.6% and 54.8%, respectively, *p* = 0.541, see Fig. 2a.

**Table 3:** Comparison of clinicopathological variables and *KRAS* mutation status in all gastric cancer cohorts combined

	*KRAS* mutation status				*p* value
	*M* *n*	*M* %	WT *n*	WT %	
Age (years)					
< 65	13	4	319	96	0.167
≥ 65	55	6	876	94	
Gender					
Male	36	5	723	95	0.225
Female	32	6	475	94	
T stage					
pT1/pT2	20	5	388	95	0.639
pT3/pT4	47	6	802	95	
N stage					
pN0	31	6	455	94	0.158
pN1–pN3	35	5	734	95	
TNM stage					
I–II	35	5	651	95	0.756
III–IV	31	6	533	95	
Lauren classification					
Diffuse	7	2	283	98	0.013
Intestinal	41	6	652	94	
Mucinous	6	12	43	88	
Indeterminate	13	6	215	94	
JGCA classification					
Pap	7	10	64	90	0.012
Tub1	7	3	212	97	
Tub2	27	7	376	93	
Por1	13	6	215	94	
Por2	6	3	219	97	
Sig	1	2	64	99	
Muc	6	12	43	88	
Morphological heterogeneity					
Homogeneous	31	6	506	94	0.550
Heterogeneous	36	5	683	95	
Microsatellite instability status					
MSI	33	17	165	83	< 0.001
MSS	32	3	1010	97	

Some variables do not add up to 1282 due to missing data

*JGCA* Japanese Gastric Cancer Association, *Pap* papillary adenocarcinoma, *Tub1* well-differentiated tubular adenocarcinoma, *Tub2* moderately differentiated tubular adenocarcinoma, *Por1* poorly differentiated adenocarcinoma solid type, *Por2* poorly differentiated adenocarcinoma non-solid type, *Sig* signet-ring cell carcinoma, *Muc* mucinous adenocarcinoma, *MSI* microsatellite instable, *MSS* microsatellite stable, *M* mutated, *WT* wild type

### *KRAS* amplification and relationship with clinicopathological variables

**Fig. 3:**
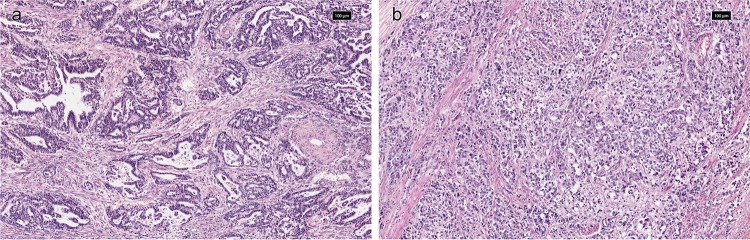
Example of *KRAS *amplified GC with **a** moderately differentiated tubular (tub2) adenocarcinoma and **b** poorly differentiated solid-type  (por1) adenocarcinoma

*KRAS* gene copy number status was available from 649 GCs (KCCH *n* = 208, LTHT *n* = 216, TCGA *n* = 225). In total, 47 (7%) GCs had a *KRAS*amp [TCGA (8%), LTHT (8%) and KCCH (6%)], see Table 2. Within *KRAS*amp GC, intestinal type (*n* = 23, 50% or tub2 (*n* = 21, 46%) was the most frequent histological phenotype by Lauren and JGCA classification, respectively (see Fig. 3a). Comparing individual histological subtypes, *KRAS*amp was more frequently found in indeterminate type (*n* = 12, 10%) or por1 (*n* = 12, 10%) phenotype by Lauren and JGCA classification, respectively (see Fig. 3b). There was no relationship between *KRAS*amp and histological phenotype or any other clinicopathological variables, see Table 4. The 5-year overall survival rate in GC patients with and without *KRAS*amp was 47.6% versus 55.6%, respectively, *p* = 0.166, see Fig. 2b.

**Table 4:** Comparison of clinicopathological variables and *KRAS* copy number status in KCCH, LTHT and TCGA gastric cancer cohorts combined

	*KRAS* amplified (*n*)	*KRAS* amplified (%)	*KRAS* other (*n*)	*KRAS* other (%)	*p* value
Age (years)					
< 65	21	8	235	92	0.462
≥ 65	26	7	364	93	
Gender					
Male	29	7	383	93	0.792
Female	18	8	219	92	
T stage					
pT1/pT2	8	7	109	93	0.867
pT3/pT4	38	7	484	93	
N stage					
pN0	7	4	163	96	0.058
pN1–pN3	40	9	428	92	
TNM stage					
I–II	14	5	262	95	0.061
III–IV	32	9	325	91	
Lauren classification					
Diffuse	10	6	168	94	0.480
Intestinal	23	7	298	93	
Mucinous	1	3	29	97	
Indeterminate	12	10	107	90	
JGCA classification					
Pap	0	0	24	100	0.267
Tub1	2	3	70	97	
Tub2	21	9	204	91	
Por1	12	10	107	90	
Por2	9	6	144	94	
Sig	1	4	24	96	
Muc	1	3	29	97	
Morphological heterogeneity					
Homogeneous	19	6	282	94	0.437
Heterogeneous	27	8	315	92	
Microsatellite instability status					
MSI	3	3	90	97	0.093
MSS	44	8	494	92	

Some variables do not add up to 822 due to missing data

*JGCA* Japanese Gastric Cancer Association, *Pap* papillary adenocarcinoma, *Tub1* well-differentiated tubular adenocarcinoma, *Tub2* moderately differentiated tubular adenocarcinoma, *Por1* poorly differentiated adenocarcinoma solid type, *Por2* poorly differentiated adenocarcinoma non-solid type, *Sig* signet-ring cell carcinoma, *Muc* mucinous adenocarcinoma, *MSI* microsatellite instable, *MSS* microsatellite stable, *KCCH* Kanagawa Cancer Center Hospital, *LTHT* Leeds Teaching Hospital Trust, *TCGA* The Cancer Genome Atlas

Only two GCs from the TCGA cohort had a concurrent *KRAS*amp and *KRAS*mut; one was a mucinous GC, the other was a por2 GC according to JGCA classification.

## Discussion

This is the largest multicentre study to date to investigate the relationship between *KRAS* activation by mutation and/or amplification and histological phenotype in GC. The frequency of *KRAS*amp (7%) was slightly higher than that of *KRAS*mut (5%) which is consistent with other GC studies [10, 11, 37]. The higher frequency of *KRAS*mut in the TCGA GC cohort compared to the other cohorts could be related to the methodology as TCGA used whole-exome sequencing to test non-hotspot regions, whereas other studies used less-sensitive Sanger sequencing/PCR–RFLP. We found *KRAS*amp and *KRAS*mut were exclusive in > 99% of GC, which is consistent with previous reports [11–13, 38].

The relationship between *KRAS*mut and histological phenotype has not been investigated in great detail and previous studies were limited by small sample sizes and hence lack of statistical power [6]. In our study, we identified a relationship between *KRAS*mut and mucinous histological phenotype, which is concordant with higher frequencies of *KRAS*mut being reported in mucinous lung [8], ovarian [9] and colorectal cancer [39, 40]. However, due to the relatively low frequency of GC with mucinous phenotype and *KRAS*mut (12%), it would not be feasible to use the presence of a mucinous phenotype as a predictor for the presence of a *KRAS*mut in GC. The main component of mucinous GCs is extracellular mucin, which consists of high molecular weight glycoproteins regulated by expression of the MUC2, MUC5AC and MUC6 genes in humans [41]. In mouse models with constitutively activated *KRAS* in the stomach, irregular MUC4+ cells were found with abnormal mucins confirmed by Alcian-blue staining [42]. Interestingly, our study suggests a relationship between *KRAS*mut and mucinous phenotype, which is characterised by extracellular mucin, but is not related to signet-ring cell type GC, which is characterised by intracellular mucin. Our study confirmed the relationship between *KRAS*mut and the presence of MSI, which our group and others have described previously in a smaller GC cohort [43, 44].

The prognostic significance of *KRAS*mut in GC remains controversial [6]. In our study, there was no association with the presence of *KRAS*mut and survival. Interestingly, in lung and colorectal cancer, *KRAS*mut has been associated with a poor prognosis [45, 46], whereas in ovarian cancer, *KRAS*mut has been associated with an improved prognosis [47].

The relationship between *KRAS*amp and clinicopathological variables, including histological phenotype in cancer is not well studied. In GC, we found no statistically significant relationship between *KRAS*amp and histological phenotype, or any other clinicopathological variables. In contrast, others found that the presence of *KRAS*amp is associated with a poor prognosis in GC [3, 10, 12]. This difference might be due to case selection and methodology used.

In our study, we used the JGCA scheme for the histological classification of GC and performed a conversion to the Lauren scheme, which is the most widely used histological classification system in Western countries [22]. Previous studies investigating the relationship between *KRAS*mut and histological phenotype performed classification according to the Lauren scheme [6], for which there is no separate category for mucinous GC. The relatively large number of GCs classified as indeterminate according to the Lauren scheme comes from conversion from the JGCA por1 histological phenotype. Direct classification according to the Lauren scheme, would likely result in a higher proportion of GCs classified as either intestinal or diffuse.

In colorectal cancer, *KRAS*mut is known to be an early event in the progression from normal colonic epithelial cell to adenoma, and finally to carcinoma [48]. The evidence of sequential development by accumulation of genetic alterations, including *KRAS*mut, is still controversial in GC [49–51]. We were unable to make any comments regarding the role of *KRAS *activation in gastric carcinogenesis in our cohort as we did not investigate precancerous lesions in the current study. However, evidence from mouse models suggest that *KRAS*mut is one of the key molecular alterations involved in the development of stomach dysplasia [52] and GC [53]. Based on the evidence from other cancer types that *KRAS*mut influence the progression of a mucinous histological phenotype, we therefore speculate based on our results, that *KRAS*mut in GC is an early event in GC development, whereas *KRAS*amp is likely to be a late event occurring after the histological phenotype has been established. This would correspond with experiments in mice expressing oncogenic *KRAS* in combination with E-cadherin and p53 loss, which resulted in a rapid progression of GC compared to wild type mice [53].

Our study has some limitations. This is a retrospective study. Histological phenotyping was performed on a single slide. Given the high frequency of intra-tumoural morphological heterogeneity in this study and the previously reported intra-tumoural heterogeneity in *KRAS*mut status in GC [54], the sensitivity of some of the techniques used in the current study may not be sufficient to detect *KRAS* activation in subclones of tumour cells. As we did not perform microdissection of tumour subregions, we cannot comment on *KRAS* status heterogeneity within the same tumour. Furthermore, we used different techniques for DNA extraction, *KRAS*mut status analysis and MSI analysis in different cohorts included in the current study, each with differing sensitivities [55, 56].

In summary, we identified a relationship between *KRAS*mut and mucinous histological phenotype in GC. The high level of intratumour morphological heterogeneity could reflect *KRAS*mut heterogeneity, which may explain the failure of anti-EGFR therapy in GC.

## Electronic supplementary material

Below is the link to the electronic supplementary material.

Supplementary file1 (DOCX 15 kb)
