# Supplementary material for: KRAS status is related to histological phenotype in gastric cancer: results from a large multicentre study
Source: Gastric Cancer. 2019 May 20;22(6):1193–203. doi: 10.1007/s10120-019-00972-6 (PMC6811379; doi:10.1007/s10120-019-00972-6)
Supplement: Supplementary file 1 — Supplementary file1 (DOCX 15 kb) [file 10120_2019_972_MOESM1_ESM.docx]

**Supplementary table 1:** Information on KRAS probes incorporated into MRC-Holland gastric cancer probemix

| **Probemix** | **Length (nucleotides)** | **Probe** | **Chromosome band** | **Exon** | **HG16 location** | **HG18 location** |
| --- | --- | --- | --- | --- | --- | --- |
| P458-A1 | 180 | 17596-L22078 | 12p12.1 | 2 | 12-025.289376 |  |
| P458-A1 | 392 | 09507-L22081 | 12p12.1 | 3 | 12-025.271583 |  |
| P458-A1 | 382 | 17605-SP0543-L21602 | 12p12.1 | 4 | 12-025.269833 |  |
| P458-A1 | 202 | 17597-SP0529-L22061 | 12p12.1 | 6 | 12-025.252102 |  |
| P458-B1 | 124 | 20117-L27312 | 12p12.1 | 6 |  | 12-025.252 |
| P458-B1 | 197 | 20095-L27280 | 12p12.1 | 4 |  | 12-025.270 |
| P458-B1 | 399 | 19323-L27531 | 12p12.1 | 3 |  | 12-025.272 |

|  |
| --- |
